# Modelling Bacteria-Inspired Dynamics with Networks of Interacting Chemicals

**DOI:** 10.3390/life9030063

**Published:** 2019-07-29

**Authors:** Tamás Bánsági, Annette F. Taylor

**Affiliations:** 1 School of Chemistry, University of Birmingham, Edgbaston B15 2TT, UK; 2 Department of Chemical and Biological Engineering, University of Sheffield, Sheffield S1 3JD, UK

**Keywords:** systems chemistry, reaction networks, autocatalysis, quorum sensing, bioinspired systems

## Abstract

One approach to understanding how life-like properties emerge involves building synthetic cellular systems that mimic certain dynamical features of living cells such as bacteria. Here, we developed a model of a reaction network in a cellular system inspired by the ability of bacteria to form a biofilm in response to increasing cell density. Our aim was to determine the role of chemical feedback in the dynamics. The feedback was applied through the enzymatic rate dependence on pH, as pH is an important parameter that controls the rates of processes in cells. We found that a switch in pH can be used to drive base-catalyzed gelation or precipitation of a substance in the external solution. A critical density of cells was required for gelation that was essentially independent of the pH-driven feedback. However, the cell pH reached a higher maximum as a result of the appearance of pH oscillations with feedback. Thus, we conclude that while feedback may not play a vital role in some density-dependent behavior in cellular systems, it nevertheless can be exploited to activate internally regulated cell processes at low cell densities.

## 1. Introduction

Bacteria are arguably one of the most prolific forms of life on earth, capable of surviving the harshest of environmental conditions. The processes that allow bacteria to maintain homeostasis, adapt to changing environmental conditions, and switch between vastly different states in response to external stimuli are often driven by internal feedback mechanisms. Feedback, when a process is regulated by its output, plays a vital role in the functioning of living organisms [1].

Feedback is also utilized to drive a sharp switch from single cell to multi-cellular behavior in a phenomenon known as quorum sensing [2]. Bacteria communicate by release and amplification of signaling species resulting in population-level responses such as the formation of biofilm above a threshold number or density [3]. Biofilms consist of a sticky matrix of polysaccharides and other biomolecules that protect the bacteria against the external environment. The development of antimicrobial resistance or tolerance typically involves the formation of a biofilm driven by a quorum sensing mechanism.

The design of biologically-relevant reaction networks that can reproduce dynamical cell behaviors may provide an insight into the role of feedback in such phenomena [4,5]. Progress in systems and synthetic biology has resulted in oscillations and quorum sensing driven by autocatalytic networks in genetically modified organisms [6]. The systems approach to chemistry is also undergoing rapid development [7] but although feedback based on DNA [8], peptides [9] and other biomolecules [10,11] has been implemented, autocatalytic communication mechanisms have been mainly investigated in regard to inorganic redox processes [12,13]. There are relatively few robust examples of chemical feedback in which cell-to-cell communication can be explored. 

Taking inspiration from bacteria, we developed a model to explore the role of feedback in a cellular system when the feedback is coupled to other process, such as gelation or precipitation of a substance in the external solution. The model consisted of an enzyme-catalyzed reaction network based on urease, a virulence factor produced by bacteria [14] and associated with biomineralization, the precipitation of inorganic salts by living organisms [15]. It has been demonstrated that the urease reaction can display feedback as a result of the enzyme rate dependence on pH [16,17]. The underlying chemistry, although biologically relevant, is not implicated in quorum sensing in bacteria. However, feedback through pH is of interest as pH plays an important role in the rates of many processes and oscillations in pH have been observed in living systems as well as implicated in the origin of life [18,19].

Here, we used the model to explore the influence of increasing density of cells on the dynamics and determined the importance of feedback on the resultant behavior. Our aim was to build a simple model that may shed light on the role of feedback in natural processes but also to aid in the design of synthetic cells that may be exploited in biotechnological applications. There have been great advances in the development of synthetic cells based on, for example, gene networks or enzymatic reactions in liposomes [20,21], but few examples exploiting feedback. Hence our model is based on an experimentally-realizable reaction network that might be implemented in a synthetic cell, rather than a realistic mimic of biological processes in bacteria. 

## 2. Methods 

The enzyme-catalyzed reaction network we considered here was inspired by the enzyme urease described in detail elsewhere [16,22], where S is the substrate and EH (active form of the enzyme) is the active form of the polyprotic enzyme which binds to the substrate to give product, N:

EH_2_^+^ ⇆ EH + H^+^ ⇆ E^−^ + 2H^+^ (1a)

EH + S ⇆ EHS ⇆ EH + N(1b)

The protonation equilibria constants of the enzyme in (1a) are *K*_e1_ and *K*_e2_. At low and high pH, the dominant forms of the enzyme are EH_2_^+^ and E^−^ respectively, which are considered inactive. Therefore, the rate of the enzyme-catalyzed reaction (1a + 1b) is given by a modified Michaelis–Menten expression, taking into account the fact that the activity depends on pH:

(1c) $$S\;\overset{\mathrm{EH}}{\to }N\;R1=\frac{v_{max}\left[S\right]}{\left(K_{M}+\left[S\right]\right)\left(1+\frac{K_{e2}}{\left[H^{+}\right]}+\frac{\left[H^{+}\right]}{K_{e1}}\right)}$$

where *v_max_* is the maximum enzyme rate and *K_M_* is the Michaelis constant. The constants for the enzyme-catalyzed reaction were *v_max_* = 0.0001 M/s; *K_e_*_1_ = 5 × 10^−6^ M; *K_e_*_2_ = 2 × 10^−9^ M and *K_M_* = 3 × 10^−3^ M. The product, N, of the enzyme reaction is a weak base, and reacts with acid to form NH^+^:

NH^+^ ⇆ N + H^+^   *R*2 = *k*_2_[NH^+^] − *k*_2r_[N][H^+^] (2)

where the net rate is *R*2 and *k*_2_ = 1 × 10^−3^ s^−1^, *k*_2r_ = 1 × 10^7^ M^−1^ s^−1^ and pK_a_ = 10. The addition of a large organic molecule, B, that can buffer changes in pH was also considered:

BH^+^ ⇆ B + H^+^   *R*3 = *k*_3_[BH^+^] − *k*_3r_[B][H^+^] (3)

with net rate *R*3 and *k*_3_ = 1, *k*_3r_ = 1 × 10^7^ and pKa = 7. 

**Figure 1:**
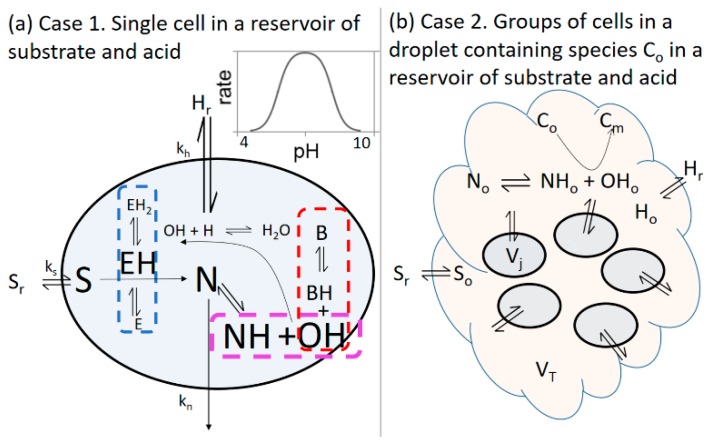
(**a**) Case 1. Reaction network and mass transfer processes used in the model of enzyme, EH, in a cell in a reservoir of constant substrate S_r_ and acid H_r_, and rate as a function of pH for the enzyme-catalyzed reaction. For simplicity, + and − charges are not shown. (**b**) Case 2. Illustration of a group of enzyme cells, each of volume *V*_j_, in an outer solution (droplet) of volume *V*_T_ in which base-catalyzed gelation/precipitation (C_o_ -> C_m_) can take place. The outer solution is connected to a constant supply of substrate S_r_ and acid H_r_ from a reservoir.

The key processes in the enzyme reaction network are illustrated in Figure 1a where the blue dashed box shows the enzyme protonation equilibria. The dependence of the enzyme rate on acid concentration gives rise to the typical bell-shaped curve expected for enzyme-catalyzed reactions, with a maximum rate for this reaction at pH = 7. Combining (2) and (3) with the water dissociation equilibrium:

H_2_O ⇆ OH^−^ + H^+^   *K*_w_ = [OH^−^][H^+^] = 1 × 10^−14^ (4)

yields the product highlighted in the purple box and the buffer equilibrium in the red dashed box. Feedback arises through the pH: starting from low pH, the production of OH^−^ leads to an increase in pH and hence an increase in enzyme rate as the reaction proceeds. The acid-dependent term in R1 was removed in simulations where no feedback was included, recovering the classic Michaelis–Menten expression relevant for buffered systems. We do not include + and − charges in the figure and the rest of the model development for simplicity.

### 2.1. Case 1: Cell in a Reservoir

In Case 1, we considered a model of a single cell containing enzyme and buffer molecules that cannot cross the cell wall (Figure 1a). The other small molecules can be transferred between the cell and the reservoir:

A_i_ ⇋ A_i_(reservoir)   rate = *k*_i_([A_i_] − [A_i,r_]) (5)

where A_i,r_ is the reservoir concentration of the *i*th species. The concentrations of S_r_ and H_r_ were taken as constant and N was considered as dilute in the reservoir such that concentrations N_r_ and NH_r_ were effectively zero. The transfer rate coefficient *k*_i_ was given by:

*k*_i_ = 3*P*_i_/*r*_j_ (6)

where *P*_i_ is the permeability coefficient (m s^−1^) that depends on the nature of the cell membrane and the species and *r*_j_ is the radius of the *j*th cell. The values of permeability coefficients vary widely. Here, in line with earlier work considering transport of species from synthetic lipid vesicles [22], we took *k*_s_ = 0.0014 s^−1^; *k*_H_ = *k*_OH_ = 0.008 s^−1^ and *k*_N_ = 0.004 s^−1^.

The rate equation for H and OH can be combined [22] and the concentration of NH determined from mass balance [NH] = [S_r_] − [S] − [N] resulting in a five variable model for case 1:

(7) $$\frac{d\left[S\right]}{dt}=-R1+k_{S}\left(\left[S_{r}\right]-\left[S\right]\right)$$

(8) $$\frac{d\left[N\right]}{dt}=R1+R2-k_{N}\left[N\right]$$

(9) $$\frac{d\left[H\right]}{dt}=\left(R2+R3+\;k_{H}\left(\left[H_{r}\right]-\left[H\right]\right)-k_{\mathrm{OH}}\left(\frac{Kw}{\left[H\right]}-\frac{Kw}{\left[H_{r}\right]}\right)\right){\left(1+\frac{Kw}{{\left[H\right]}^{2}}\right)}^{-1}$$

(10) $$\frac{d\left[B\right]}{dt}=R3$$

(11) $$\frac{d\left[\mathrm{BH}\right]}{dt}=-R3$$

### 2.2. Case 2: Group of Identical Cells in a Fixed Volume of Solution

In the second case, we considered a scenario where a group of cells had secreted a substance, C, which accumulated in a droplet around the cells, where [C_o_] is the concentration of C in the droplet. The cells of volume *V*_j_ were contained in a fixed volume, *V*_T_, of outer solution (the droplet) in which base-catalyzed gelation or precipitation of C_o_ can take place (Figure 1b):

C_o_(soluble) → C_m_(insoluble)   *R*12 = *k*_12_[C_o_][OH^−^] (12)

with an empirical rate *R*12. The concentrations [C_o_] and [C_m_] were calculated in the model in units of mol/dm^3^ as they corresponded to the total amount (moles) of C present in free form, C_o_, compared to the amount (moles) of C in precipitated or gelled form, C_m_, in the droplet. The extent of conversion was obtained from *p*_CM_ = [C_m_]/([C_m_] + [C_o_]). The reverse process was considered negligible on the timescales investigated here and uniform concentrations were assumed in the outer solution. The value of *k*_12_ was taken as 100 M^−1^ s^−1^.

The outer solution was connected to a larger reservoir of fixed substrate and acid concentration, S_r_ and H_r_. The rate of change of concentration of the species, S, H, N and NH in the outer solution was given by the relevant equilibria, net rate of transfer from the reservoir and net rate of transfer from the cells, which is calculated from the sum of the moles of species transferred from each cell per unit time, divided by the volume, *V*_T_, of outer solution. For j = 1…*n* cells:

(13) $$\frac{d\left[A_{i,o}\right]}{dt}=g\left(\left[A_{i,o}\right]\right)+k_{r}\left([A_{i,r}]-[A_{i,o}]\right)+\frac{1}{V_{T}}\overset{n}{\underset{j}{\sum }}V_{j}k_{i}\left(\left[A_{i}\right]-[A_{i,o}\right])$$

where [A_i,o_] is the concentration of the *i*th species in the outer solution, *g*([A_i,o_]) contains reaction terms, *k*_r_ is the transfer rate from the reservoir and A_i,r_ is the concentration of the *i*th species in the reservoir. For *n* identical cells the outer solution concentration is given by:

(14) $$\frac{d[A_{i,o}]}{dt}=g\left(\left[A_{i,o}\right]\right)+k_{r}\left(\left[A_{i,r}]-[A_{i,o}\right]\right)+dk_{i}\left(\left[A_{i}\right]-[A_{i,o}\right])$$

where *d* = *nV*_j_/*V*_T_ is a cell volume fraction: it gives the change in concentration of the species upon transfer from the cells of total volume *nV*_j_ to the large outer volume *V*_T_. The rate equations for the outer solution concentrations were given by:

(15) $$\frac{d\left[S_{o}\right]}{dt}=k_{r}\left(\left[S_{r}\right]-\left[S_{o}\right]\right)+dk_{S}\left(\left[S\right]-\left[S_{o}\right]\right)$$

(16) $$\frac{d\left[N_{o}\right]}{dt}=R2_{o}+\;dk_{N}\left(\left[N\right]-\left[N_{o}\right]\right)$$

(17) $$\frac{d\left[{\mathrm{NH}}_{o}\right]}{dt}=-R2_{o}+\;dk_{N}\left(\left[\mathrm{NH}\right]-\left[{\mathrm{NH}}_{o}\right]\right)$$

(18) $$\frac{d\left[H_{o}\right]}{dt}=\left(k_{r}\left(\left[H_{r}]-[H_{o}\right]\right)+R2o+\;dk_{H}\left(\left[H\right]-\left[H_{o}\right]\right)-dk_{\mathrm{OH}}\left(\frac{Kw}{\left[H\right]}-\frac{Kw}{\left[H_{o}\right]}\right)\right){\left(1+\frac{Kw}{{\left[H_{o}\right]}^{2}}\right)}^{-1}$$

(19) $$\frac{d\left[C_{o}\right]}{dt}=-R12$$

(20) $$\frac{d\left[C_{m}\right]}{dt}=R12$$

where *R*2_o_ is the net rate of reaction 2 with the concentrations of N_o_ and NH_o_ in the outer solution. We ignore the loss of N_o_ and NH_o_ to the reservoir as these were found to have negligible effect on the dynamics. Equations (15–20) were coupled with rate equations for the species S, H, N, NH, B and BH in the cells, resulting in a twelve variable model for Case 2. 

All equations were solved using XPP-AUT with the integration method of cvode [23]. Bifurcation diagrams were produced using the AUTO program in XPP. The “.ode” files are available in the supplementary information.

## 3. Results

In the enzyme-catalyzed reaction considered here, feedback was imposed through the dependence of the enzyme activity on the pH. The role of the feedback in the dynamics displayed in a single and multi-cell system connected to an external solution is explored below. 

### 3.1. Case 1: Cell in a Reservoir

**Figure 2:**
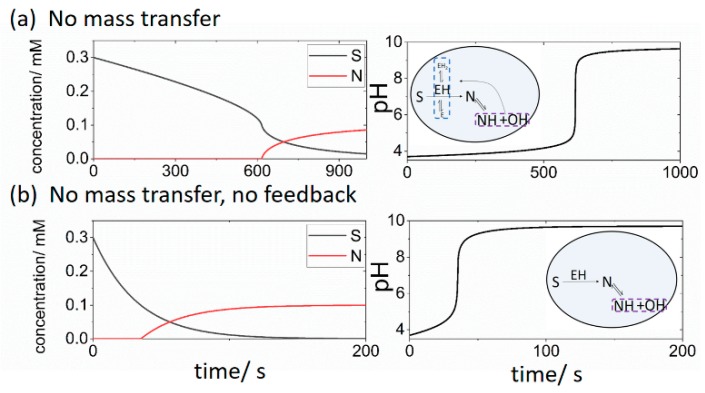
Changes in concentrations of substrate S and product N (left) and pH (right) in a closed cell with the initial [S] = 0.3 mM, [BH + B] = 0 mM, pH = 3.7, *k*_i_ = 0 and (**a**) with feedback (pH dependent *R*1) (**b**) without feedback (pH independent *R*1). Insets illustrate processes considered in the cell model.

In a cell with initial substrate [S] = 0.3 mM, no buffer, pH = 3.7 and no mass transfer between the cell and reservoir we observed a lag of approximately 600 s before the appearance of basic product N (Figure 2a). The pH switched from 4 to 9 and was accompanied by an acceleration in the rate of consumption of substrate S. In Figure 2b, no feedback was present i.e., the terms in *R*1 that depended on H were not included, and the initial concentrations were the same as in Figure 2a. The reaction was faster and there was the sudden appearance of product N after a time lag of 40 s accompanied by a pH switch from low to high. There was no acceleration in the rate of loss of substrate S when the pH rapidly increased. 

**Figure 3:**
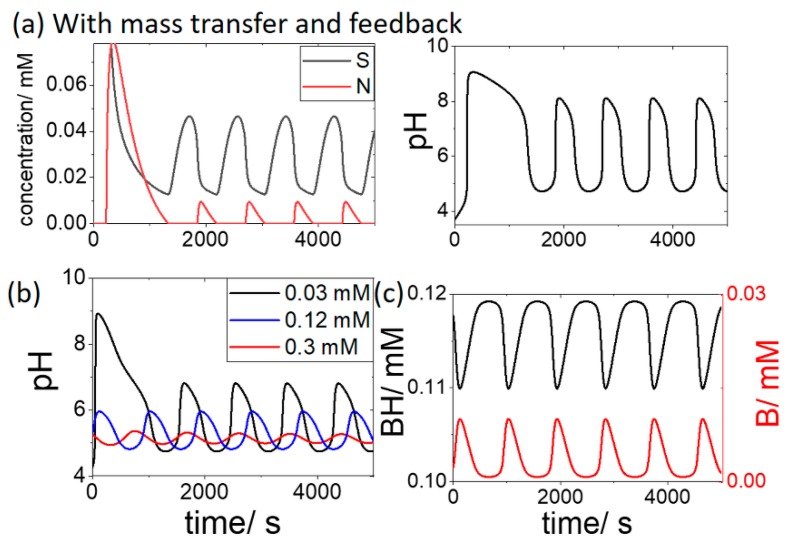
Oscillations in the open cell with substrate [S_r_] = 0.3 mM, pHr = 4.3 and effect of buffer on the oscillations where (**a**) [BH + B] = 0 mM. (**b**) initial [BH] = 0.03 mM (black), 0.12 mM (blue) and 0.3 mM (red) and (**c**) initial [BH] = 0.12 mM showing variations of BH and B in time.

In the latter two examples the reaction proceeded until all of the initial substrate was consumed. With the addition of mass transfer between the cell and the external reservoir, oscillations in substrate, product and pH were observed (Figure 3a). The transport of OH and N played a small role in the dynamics: setting the values of *k*_OH_ or *k*_N_ to zero resulted in only a slight change in the period and amplitude of the oscillations. However, oscillations occurred for a narrow range of values of the ratio of *k*_H_/*k*_S_ (5 to 6.67) in agreement with earlier work [22]. 

The influence of the addition of the buffer (BH + B) on the oscillations is shown in Figure 3b. With an initial [BH] of 0.03 mM, a reduction of the pH maximum was observed, from pH 8 to 7. When the BH concentration was increased above the substrate concentration of 0.3 mM, the pH oscillations were suppressed. The oscillations in the concentration of the buffer components in time is shown in Figure 3c for the case where initial [BH] = 0.12 mM. 

**Figure 4:**
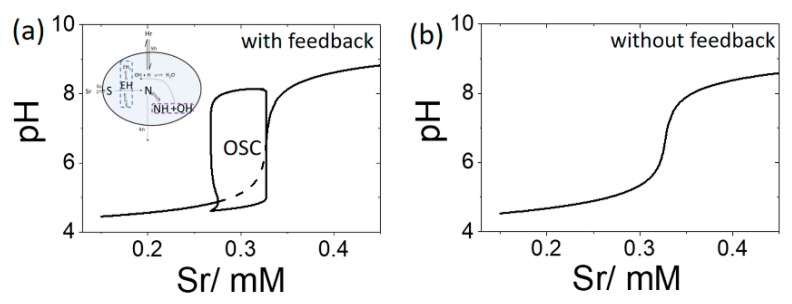
Bifurcation diagrams depicting cell pH as a function of substrate [S_r_] with pH_r_ = 4.3 and [BH] = [B] = 0 mM (**a**) enzyme rate R1 includes pH dependence, OSC shows region of oscillations with values of pH min and max indicated and dotted line shows unstable steady state (**b**) enzyme rate *R*1 does not include pH dependence, a single value of pH is observed for all S_r_.

A bifurcation diagram is plotted in Figure 4a for the open system showing cell pH as function of substrate concentration in the reservoir, [S_r_], with pH_r_ = 4.3 and [BH + B] = 0 mM. Oscillations occurred for a range of substrate values from 0.27 mM to 0.33 mM. Outside of this region, a low pH steady state (pH 4) was observed at low substrate concentrations, and a high pH steady state (pH 9) was obtained at high substrate. If the feedback term in *R*1 was removed, then no oscillations were observed for any values of S_r_ (Figure 4b). 

### 3.2. Case 2. Group of Cells in a Fixed Volume of Solution

In case 2, *n* identical cells were coupled to an outer solution (droplet) of fixed volume *V*_T_ in which the pH can change and a base-catalyzed gelation or precipitation of species C took place. The effect of increasing the value of *d = nV*_j_*/V*_T_ on the dynamics was explored. Assuming the cells have constant volume, *V*_j_, then an increase in *d* corresponded to an increase in the number density of cells. 

**Figure 5:**
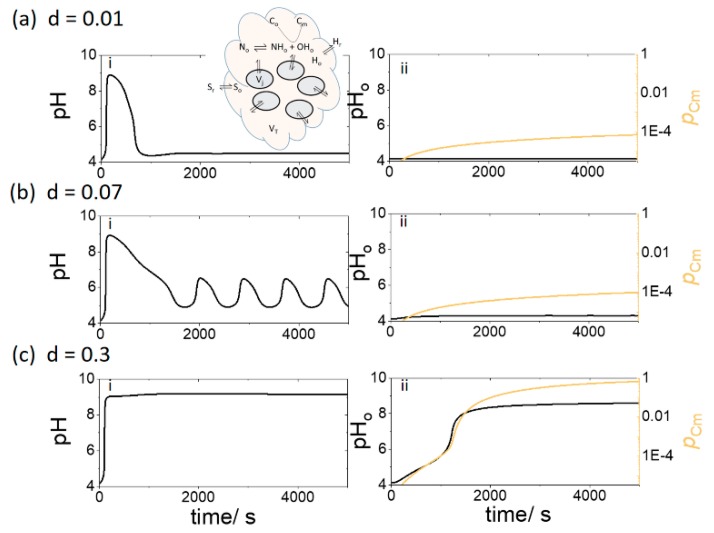
Gelation in outer solution of fixed volume, V_T,_ with an increasing density of cells (through *d*, where *d = nV*_j_*/V*_T_) with pH_r_ = 4.1, [S_r_] = 0.3 mM, [BH] = 0.03 mM and [C_o_] = 3 mM. (i) pH in enzyme cells and (ii) pH_o_ and extent of conversion of C, *p*_Cm_, in outer solution where *d* = (**a**) 0.01; (**b**) 0.07 (**c**) 0.3.

In Figure 5a, with *d* = 0.01 and [S_r_] = 0.3 mM and pH_r_ = 4.1, the internal cell pH rose initially but fell to a low pH as a result of transport of acid from the surrounding solution. The outer solution pH did not rise above pH 4 and the extent of conversion (p_Cm_) of C to gelled form, C_m_, remained low. When the number density of cells was increased (*d* = 0.07), the cell pH oscillated between pH 4.5 and 6.5 (Figure 5b). However, the outer solution pH_o_ remained low and no significant gelation was observed. If the density of cells was further increased (*d* = 0.3), then the cell pH rapidly jumped to pH 9 and the outer solution pH_o_ rose after a lag time (Figure 5c). The increase in pH_o_ catalyzed the gelation process and there was complete conversion of the soluble species C_o_ to insoluble gel C_m_. 

**Figure 6:**
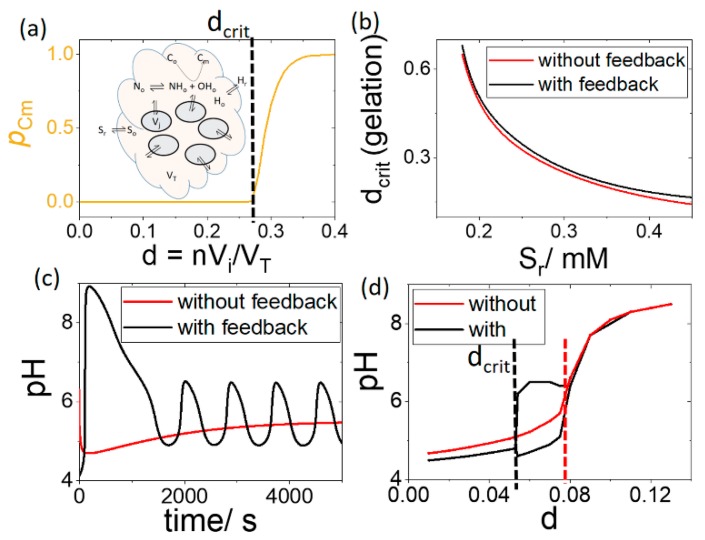
Influence of feedback on pH and gelation in group of cells in solution with pH_r_ = 4.1, [S_r_] = 0.3 M, [BH] = 0.03 mM and [C_o_] = 3 mM. (**a**) Extent of conversion, *p*_Cm_, as a function of increasing number of cells or number density *d* indicating the critical value of *d* for gelation (**b**) *d*_crit_ for gelation with feedback (pH dependent R1) and without feedback (pH independent R1). (**c**) cell pH as a function of time with feedback and without feedback for *d* = 0.07 and (**d**) cell pH as a function of *d* indicating the critical value of *d* for pH > 6.

In Figure 6a, the extent of conversion, *p*_Cm_, at 5000 s was plotted as a function of increasing number density of cells. There was a critical value of *d* above which gelation was observed in the outer solution. Below *d*_crit_, the value of C_m_ increased at such a low rate that no net gelation was obtained. The value of *d*_crit_ is plotted as a function of [S_o_] with and without feedback in Figure 6b, i.e., when the rate of the enzyme reaction *R*1 does not depend on acid. It is clear that feedback did not influence the pH switch and gelation in the outer solution. 

The feedback nevertheless played an important role in the internal cell dynamics. As *d* was increased, oscillations appeared resulting in a higher pH maximum with feedback than without (Figure 6c). The cell pH is plotted as a function of *d* in Figure 6d, where the black line shows the upper and lower values of pH in the oscillatory regime. For the pH to reach values above 6, the critical value of *d* required with feedback is almost half of that without feedback (Figure 6d dashed lines). 

## 4. Discussion

Feedback plays a vital role in the functioning of living systems [1]. One of the important applications of feedback in bacteria is the release and detection of a small diffusible molecule, the autoinducer, in a phenomenon known as quorum sensing [24]. Quorum sensing is used by bacteria to activate population-level responses such as the formation of biofilm and a transition from single cell to multi-cellular behavior [3]. In biofilms, cells are bound together in a matrix of polysaccharides and other biomolecules that protect them from the external environment. Here, inspired by biofilm formation, we have developed a reaction network in a cellular system coupling feedback with other chemical processes such as gelation in the external solution. We chose to examine feedback through pH because pH is an important parameter that controls the rates of many processes. Our system is also based on experimentally-realizable processes that could be implemented in a synthetic cell.

In our simple model, the enzyme reaction took place in a cell coupled to an external solution containing substrate, S, and acid. Feedback arose through the characteristic bell-shaped rate-pH curve of the enzyme-catalyzed reaction. The reaction produced a weak base and the rate of the enzyme reaction depended on the pH, with a maximum at pH 7. In a cell with no external supply of species, we obtained a switch from low pH to high pH after a time lag accompanied by the sudden appearance of product, N, and rapid consumption of substrate. This is an example of pH-driven feedback with the basic product of the enzyme reaction acting as autocatalyst. 

If the feedback was removed by making the enzyme rate independent of pH then we observed no rate acceleration in the loss of substrate. Nevertheless, the basic product, N, suddenly appeared after a time lag and there was a switch to high pH. In this acid-base reaction system, the initial acid reacted with the base and kept the pH low until sufficient base was produced to overcome the acid. This is similar to the substrate-depletive chemical clock reactions driven by inhibitor removal in the absence of feedback i.e., acid acted as an inhibitor suppressing formation of OH^−^ [25].

When the mass transport of species from an external reservoir of constant concentrations was included, we obtained oscillations in pH. The differential transport of acid and substrate was essential for the observation of oscillations, i.e., the transport rate constant *k*_H_ > *k*_S_. This is in agreement with earlier work on pH oscillations in some enzyme-catalyzed reactions [26,27,28]. Oscillations typically require positive feedback coupled with delayed negative feedback. The net rate of the transport of acid into the cell increased with increasing difference between the inner and outer pH, i.e., as the pH increased the rate of transport of acid into the cell increased. Therefore, the acid provided the necessary delayed negative feedback that terminated the autocatalytic, base-producing process. 

There are few experimental examples of transport-driven instabilities in chemical systems, where the differential rates of transport of species cause oscillations in space or time [29]. The concept was first explored in Turing’s pioneering modelling work on the basis of morphogenesis, in which spatial patterns were observed in a ring of coupled cells when the inhibitor transport was greater than that of the substrate in an autocatalytic process [30]. Differential transport may arise naturally in acid-base systems as acid, in the form H_3_O+, diffuses typically much quicker than other small ions in solution: the diffusion coefficient of acid can be up to nine times greater than small ions. In the model presented here, the chemical species were produced in a cell and crossed the cell wall with rates that were related to the permeability coefficients and radius of the cell. Permeability coefficients vary widely and depend on the nature of the membrane components, membrane thickness as well as the species involved [31]. In general, charged species do not cross cell membranes with appreciable rates. However, in a synthetic cell system constructed with phospholipids, fast acid transport could be facilitated by the incorporation of ion channels in the cell membrane [32]. 

Living cells are filled with buffering agents, so it is important to understand the influence that buffers might have on pH-driven feedback. We found that oscillations were, not surprisingly, completely suppressed for values of the buffer concentration greater than the substrate concentration. However, for lower concentrations the buffer primarily reduced the maximum of the oscillations. Thus, buffers could be used to control the amplitude of oscillations with a pH maximum less than 7. 

We mapped the behavior of the single cell using bifurcation diagrams of pH as a function of substrate concentration in the reservoir, [S_r_]. The transition from a low pH state to oscillations was obtained with feedback whereas only a single steady state pH was observed when no feedback was present. Nevertheless, we obtained a sharp switch in pH as [S_r_] was increased in both cases. Thus the system may be considered ultrasensitive to small changes in substrate, even in the absence of feedback [1]. The acid acting as an inhibitor played an important role in generating this response. 

In the model with multiple cells, we demonstrated that the increase in pH could be used to drive a base-catalyzed gelation or precipitation process in the external solution. This process was inspired by the quorum sensing of bacteria that leads to the formation of biofilm containing polysaccharides or the precipitation of inorganic minerals such as calcium carbonate, which occur in natural systems as the pH of the solution increases. We found that above a threshold density of cells, *d* = 0.3, the pH of the external solution increased, and gelation/precipitation occurred. A value of *d* = 0.01 corresponds to a number density of 2.7 million cells per ml of solution, assuming cells of radius 1 µm; comparable with bacterial cell cultures. The critical density of cells for a switch to high pH and gel formation was not influenced by the presence of feedback, rather the inhibition by acid played the major role in this switch-like behavior. 

We note that the behavior here was obtained for relatively low transfer rates in which a decoupling between the internal and external system was observed: there was a difference in the timescales of the pH dynamics in the cell and the pH dynamics in the outer solution. No large amplitude oscillations in pH in the external environment were obtained for any values of the parameters explored. The transfer coefficients ranged from 0.0014–0.008 s^−1^; with a cell of the order 1 μm, this gives permeability coefficients from 10^−9^–10^−8^ m s^−1^, on the low side for transport of neutral molecules in synthetic vesicle systems. It may be that for larger transport coefficients the feedback plays a greater role in the switch for gelation. However, we demonstrated that the feedback played an important role in the internal cell dynamics. The emergence of oscillations above a critical density of cells resulted in a much higher cell pH than without feedback. Thus, we conclude that the presence of feedback could be used to drive an internal switch that activates other pH-regulated processes within the cell at low substrate or cell densities. 

## 5. Conclusions

The design of reaction networks that mimic the feedback-driven behavior of biological systems remains a formidable challenge. There has been increasing interest in obtaining quorum sensing type behavior in synthetic systems [5,12,33,34,35,36,37]. The underlying processes are vastly different however all examples exploit seemingly similar principles of an autocatalytic signaling process and communication between entities via a common surround. Here, taking inspiration from bacteria, we have proposed a model to determine the role of feedback in certain density-dependent behavior. The model involved an enzyme catalyzed reaction in cells which influenced the pH and the dynamics of an external pH-regulated process: the base-catalyzed formation of a gel or precipitate. 

We found that a sharp switch in pH was obtained in the multi-cellular system above a critical number of cells and gelation/precipitation did not require the presence feedback. However, we only observed pH oscillations with feedback and the oscillations resulted in a higher pH maximum in the cells. We conclude that feedback may be used to activate internal cellular processes at low substrate concentrations and low cell densities, even when it plays no role in the external dynamics. Although the chemistry of the underlying feedback processes was not necessarily relevant to quorum sensing, similar dynamical responses may arise in cells. This model system was also based on processes that might be used for synthetic quorum sensing analogues in biotechnology applications [38,39].

## Supplementary Materials

The following are available online at https://www.mdpi.com/2075-1729/9/3/63/s1, XPP ode file for model case 1 and model case 2.

## References

[1] Tyson J.J., Chen K.C., Novak B. (2003). Sniffers, buzzers, toggles and blinkers: Dynamics of regulatory and signaling pathways in the cell. Curr. Opin. Cell Biol..

[2] Waters C.M., Bassler B.L. (2005). Quorum sensing: Cell-to-cell communication in bacteria. Annu. Rev. Cell Dev. Biol..

[3] Davies D.G., Parsek M.R., Pearson J.P., Iglewski B.H., Costerton J.W., Greenberg E.P. (1998). The involvement of cell-to-cell signals in the development of a bacterial biofilm. Science.

[4] Semenov S.N., Kraft L.J., Ainla A., Zhao M., Baghbanzadeh M., Campbell V.E., Kang K., Fox J.M., Whitesides G.M. (2016). Autocatalytic, bistable, oscillatory networks of biologically relevant organic reactions. Nature.

[5] Niederholtmeyer H., Chaggan C., Devaraj N.K. (2018). Communication and quorum sensing in non-living mimics of eukaryotic cells. Nat. Commun..

[6] Danino T., Mondragón-Palomino O., Tsimring L., Hasty J. (2010). A synchronized quorum of genetic clocks. Nature.

[7] Ashkenasy G., Hermans T.M., Otto S., Taylor A.F. (2017). Systems chemistry. Chem. Soc. Rev..

[8] Padirac A., Fujii T., Estévez-Torres A., Rondelez Y.J. (2013). Spatial waves in synthetic biochemical networks. Am. Chem. Soc..

[9] Mukherjee R., Cohen-Luria R., Wagner N., Ashkenasy G. (2015). A Bistable Switch in Dynamic Thiodepsipeptide Folding and Template-Directed Ligation. Angew. Chem. Int. Ed..

[10] Míguez D.G., Vanag V.K., Epstein I.R. (2007). Fronts and pulses in an enzymatic reaction catalyzed by glucose oxidase. Proc. Natl. Acad. Sci. USA.

[11] Bodó G., Branca R.M.M., Tóth Á., Horváth D., Bagyinka C. (2009). Concentration-dependent front velocity of the autocatalytic hydrogenase reaction. Biophys. J..

[12] Taylor A.F., Tinsley M.R., Wang F., Huang Z., Showalter K. (2009). Dynamical quorum sensing and synchronization in large populations of chemical oscillators. Science.

[13] Epstein I.R., Xu B. (2016). Reaction–diffusion processes at the nano-and microscales. Nat. Nanotechnol..

[14] Krajewska B. (2009). Ureases I. Functional, catalytic and kinetic properties: A review. J. Mol. Catal. B Enzym..

[15] Phillips A.J., Gerlach R., Lauchnor E., Mitchell A.C., Cunningham A.B., Spangler L. (2013). Engineered applications of ureolytic biomineralization: A review. Biofouling.

[16] Hu G., Pojman J.A., Scott S.K., Wrobel M.M., Taylor A.F.J. (2010). Base-Catalyzed Feedback in the Urea− Urease Reaction. Phys. Chem. B.

[17] Bánsági T., Taylor A.F. (2018). Switches induced by quorum sensing in a model of enzyme-loaded microparticles. J. R. Soc. Interface.

[18] Keil L.M.R., Möller F.M., Kieß M., Kudella P.W., Mast C.B. (2017). Proton gradients and pH oscillations emerge from heat flow at the microscale. Nat. Commun..

[19] Ball R., Brindley J. (2015). The life story of hydrogen peroxide II: A periodic pH and thermochemical drive for the RNA world. J. R. Soc. Interface.

[20] Stano P. (2019). Is Research on “Synthetic Cells” Moving to the Next Level?. Life.

[21] Küchler A., Yoshimoto M., Luginbühl S., Mavelli F., Walde P. (2016). Enzymatic reactions in confined environments. Nat. Nanotechnol..

[22] Bánsági T., Taylor A.F.J. (2014). Role of differential transport in an oscillatory enzyme reaction. Phys. Chem. B.

[23] Ermentrout B. (2002). Simulating, Analyzing, and Animating Dynamical Systems.

[24] Miller M.B., Bassler B.L. (2001). Quorum sensing in bacteria. Annu. Rev. Microbiol..

[25] Horváth A.K., Nagypál I. (2015). Classification of clock reactions. ChemPhysChem.

[26] Caplan S.R., Naparstek A., Zabusky N.J. (1973). Chemical oscillations in a membrane. Nature.

[27] Miele Y., Bánsági T., Taylor A.F., Stano P., Rossi F. (2016). Advances in Artificial Life, Evolutionary Computation and Systems Chemistry: 10th Italian Workshop, WIVACE 2015, Bari, Italy, 22–25 September 2015.

[28] Vanag V.K., Míguez D.G., Epstein I.R. (2006). Designing an enzymatic oscillator: bistability and feedback controlled oscillations with glucose oxidase in a continuous flow stirred tank reactor. J. Chem. Phys..

[29] Horváth J., Szalai I., De Kepper P. (2009). An experimental design method leading to chemical Turing patterns. Science.

[30] Turing A.M. (1952). The chemical basis of morphogenesis. Phil. Trans. R. Soc. Lond. Ser. B.

[31] Paula S., Volkov A.G., Van Hoek A.N., Haines T.H., Deamer D.W. (1996). Permeation of protons, potassium ions, and small polar molecules through phospholipid bilayers as a function of membrane thickness. Biophys. J..

[32] Fyles T.M. (2007). Synthetic ion channels in bilayer membranes. Chem. Soc. Rev..

[33] Garcia-Ojalvo J., Elowitz M.B., Strogatz S.H. (2004). Modeling a synthetic multicellular clock: Repressilators coupled by quorum sensing. Proc. Natl. Acad. Sci. USA.

[34] Shum H., Balazs A.C. (2017). Synthetic quorum sensing in model microcapsule colonies. Proc. Natl. Acad. Sci. USA.

[35] Singh H., Parmananda P. (2013). Quorum sensing via static coupling demonstrated by Chua’s circuits. Phys. Rev. E.

[36] Szabo E. (2015). Oregonator generalization as a minimal model of quorum sensing in Belousov–Zhabotinsky reaction with catalyst confinement in large populations of particles. RSC Adv..

[37] Zamora-Munt J., Masoller C., Garcia-Ojalvo J., Roy R. (2010). Crowd synchrony and quorum sensing in delay-coupled lasers. Phys. Rev. Lett..

[38] Din M.O., Danino T., Prindle A., Skalak M., Selimkhanov J., Allen K., Julio E., Atolia E., Tsimring L.S., Bhatia S.N. (2016). Synchronized cycles of bacterial lysis for in vivo delivery. Nature.

[39] Tóth-Szeles E., Horváth J., Holló G., Szűcs R., Nakanishi H., Lagzi I. (2017). Chemically coded time-programmed self-assembly. Mol. Syst. Des. Eng..

