# Supplementary material for: Modelling Bacteria-Inspired Dynamics with Networks of Interacting Chemicals
Source: Life (Basel). 2019 Jul 29;9(3):63. doi: 10.3390/life9030063 (PMC6789575; doi:10.3390/life9030063)
Supplement: Supplementary file 1 [file life-09-00063-s001.pdf]

Modelling bacteria-inspired dynamics with networks of interacting chemicals

Tamás Bánsági Jr,[1,2] and Annette F. Taylor [2,*]

Supplementary information

The ODE files that were used in case 1 and case 2 are included below. All equations were solved using XPP-AUT with the integration method of cvode.[1] Bifurcation diagrams were produced using the AUTO program in XPP.

Model 1 .ode file for case 1: cell in a reservoir

```
#5 variable enzyme hydrolysis with scalings S(units)=KM*s; H=KE1*h; N=KM*n; B=KM*b; BH=KM*bh

#rates
R1 = vmax*s/((1+l*Ke2/(Ke1*h)+l*h)*(1+s))
R2 = k2r*KM*(sr-s-n)-k2*n*h*KM*Ke1
R3 = k3*KM*bh-k3r*b*h*KM*Ke1

#rate equations
s'=-R1/KM+ks*(sr-s)
n'=R1/KM+R2/KM-kn*n
h'=(R2/Ke1+kh*(hr-h)+R3/Ke1-koh*(Kw/hr-Kw/h)/(Ke1*Ke1))/(1+KW/(h*h*Ke1*Ke1))
#buffer - remove for bifurcation diagram
b'=R3/kM
bh'=-R3/kM

aux pH=-log(Ke1*h)/log(10)

#parameters
par vmax=1e-4,KM=0.003,Ke1=5e-6,Ke2=2e-9,k2r=1e-3,k2=1e7,Kw=1e-14,k3=1, k3r=1e7
par l=1
par ks=0.0014,kh=0.008,koh=0.008,kn=0.004,sr=0.1,hr=11

#initial conditions - scaled!
init s=0.1,h=11

#numerical stuff
@ total=5000,dt=0.1,tol=1e-12, atol=1e-8, meth=cvode
@ xplot=t,yplot=pH,xhi=5000,ylo=1,yhi=14
@ maxstor=10000000
Done
```

Model 2 .ode file for case 2: Group of identical cells in a fixed volume of solution

```
#12 variable enzyme hydrolysis with scalings: S(units)=KM*s; H=KE1*h; N=KM*n; B=KM*b;
BH=KM*bh; Co = KM*co

#rates in the cells
R1 = vmax*s/((1+l*ke2/(ke1*h)+l*h)*(1+s))
R2 = k2*KM*nh-k2r*n*h*KM*Ke1
R3 = k3*KM*bh-k3r*b*h*KM*Ke1

#rate equations
s'=-R1/KM+ks*(so-s)
n'=R1/kM+R2/KM+kn*(no-n)
h'=(R2/Ke1+R3/Ke1+kh*(ho-h)-kh*(Kw/ho-Kw/h)/(Ke1*Ke1))/(1+Kw/(h*h))
nh'=-R2/KM+kn*(nho-nh)
b'=R3/KM
bh'=-R3/KM

#rates in the droplet
R2o=k2*KM*nho-k2r*no*no*KM*Ke1
R4o=kc*Kw/(ho*Ke1)*co*KM

#rate equations
so'=kr*(sr-so)+ks*d*(s-so)
no'=R2o/KM+kn*d*(n-no)
ho'=(kr*(hr-ho)+R2o/ke1+kh*d*(h-ho)-kh*d*(Kw/h-Kw/ho)/(Ke1*Ke1))/(1+Kw/(ho*ho))
nho'=-R2o/kM+kn*d*(nh-nho)
co'=-R4o/KM
cm'=R4o/KM

aux pH=-log(Ke1*h)/log(10)
aux pHo=-log(Ke1*ho)/log(10)

#parameters
par sr=0.1,hr=15,vmax=1e-4,KM=0.003,Ke1=5e-6,Ke2=2e-9,k2=1e-3,k2r=1e7,Kw=1e-14
par kr=0.001,k3=1, k3r=1e7, kc=100
par l=1,d=0.07
par ks=0.0014,kh=0.008,kn=0.004

#initial conditions - scaled!
init s=0.1,h=15,ho=15,so=0.1,bh=0.01,co=1


#numerical stuff
@ total=5000,dt=1,tol=1e-12, atol=1e-8, meth=cvode
@ xplot=t,yplot=pH,xhi=5000,ylo=1,yhi=14
@ maxstor=10000000, bounds=10000
done
```


1. B. Ermentrout, *Simulating, Analyzing, and Animating Dynamical Systems*. (Society for Industrial and Applied Mathematics, 2002).
